# Mindfulness-based stress reduction for autistic adults: A feasibility study in an outpatient context

**DOI:** 10.1177/13623613231172809

**Published:** 2023-05-16

**Authors:** Hanna Agius, Anne-Kristina Luoto, Anna Backman, Carina Eriksdotter, Nitya Jayaram-Lindström, Sven Bölte, Tatja Hirvikoski

**Affiliations:** 1 Karolinska Institutet, Stockholm Health Care Services, Sweden; 2 Stockholm Health Care Services, Sweden; 3 Curtin University, Australia

**Keywords:** adults, autism, intervention, mindfulness, stress

## Abstract

Autistic adults report high stress and impaired ability to cope with stressors. Mindfulness-based stress reduction targets individual’s own resources to regulate stress responses. An open feasibility study was conducted in an outpatient context, recruiting autistic adults without intellectual disability. The intervention, guided by trained mindfulness-based stress reduction teachers, followed the contents of the standard manual while adapting the delivery to autistic adults’ needs. The total sample comprised of *N* = 50 participants; *N* = 43 (86%) attended at least one session, and *N* = 34 of these (79%) completed the intervention. Participants found mindfulness-based stress reduction logical, likely to lead to improvement and recommendable to autistic peers. No serious adverse events were reported. Measures of preliminary effectiveness indicated reduced symptoms of stress (*p* < 0.001; *d* = 0.51) and improved stress-coping skills (*p* = 0.017; *d* = 0.43) from pre- to post-intervention, as well as a reduction in symptoms of anxiety and depression. No changes were observed in mindful attention awareness, life satisfaction, or acceptance of the autism diagnosis. The data suggest that mindfulness-based stress reduction is a feasible and acceptable intervention for autistic adults in an outpatient setting. Further investigation in randomized clinical trials is warranted (Clinical Trials, clinicaltrials.gov, registration no. NCT05244265).

**Lay abstract:**

Autistic adults report high stress levels and difficulties dealing with everyday stressors. Mindfulness-based stress reduction groups aim to help regulate stress responses. We asked 50 autistic adults, without intellectual disability, to participate in a study of mindfulness-based stress reduction. The group program was made accessible through clear group leader communication and good program predictability, as well as reduced exposure to disturbing sensory stimuli. The mindfulness and yoga based exercises from the original mindfulness-based stress reduction program were included. The participants were positive and would even recommend an autistic friend to participate in a mindfulness-based stress reduction group. They reported that mindfulness-based stress reduction could lower symptoms of stress and improved stress coping. We still need to investigate these effects further in larger studies. The findings of this work show that mindfulness-based stress reduction groups can be adapted for autistic adults and that the participants overall were positive to the intervention and the group format.

Autism spectrum disorder (henceforth, autism) is a persistent neurodevelopmental condition (American Psychiatric Association, 2013). Transitioning to adulthood, building, and sustaining an independent life is often challenging for autistic people (Howlin, 2021; Jonsson et al., 2021; Mason et al., 2021), causing long-term stress that may contribute to the poor mental health observed among autistic adults (Hirvikoski et al., 2020; Hollocks et al., 2019). However, autistic adults report stress not only related to life domains (housing, work, and relationships) but also to the lack of tangible support and treatment (Moseley et al., 2021), underlining the importance of preventive resilience-building interventions. Emerging evidence suggests that mindfulness-based interventions may enhance stress regulation and coping with stress in autistic adults (Hourston & Atchley, 2017; White et al., 2018).

Autistic individuals experience both more stress in everyday life (Bishop-Fitzpatrick et al., 2018; Hirvikoski & Blomqvist, 2015) and more stressful major life events than non-autistic adults (Moseley et al., 2021). The impact of both daily hassles and major life events depends on the individual’s internal (e.g. coping skills) and external (e.g. social support) resources to face the stressors (Karasek, 1979; Lazarus & Folkman, 1984). Autistic adults report poorer ability to use coping strategies to manage daily stressors (Hirvikoski & Blomqvist, 2015), and social support as a coping strategy may be limited by social interaction difficulties (Chan et al., 2022). Stressors related to health care and other services (Moseley et al., 2021) may be associated both with poor accessibility, such as long wait times (Adams & Young, 2021), and with lack of sufficient knowledge on autism among professionals in these diverse settings (Maddox & Gaus, 2019). These barriers in health care encounters could exacerbate the symptoms of stress further, while interventions providing adaptive coping skills could prevent long-term stress from developing into conditions that require psychiatric services in the first place (Bishop-Fitzpatrick et al., 2017; Mandy, 2022; Moseley et al., 2021; White et al., 2018). Such interventions must, however, be acceptable for autistic adults, including adjusting how the treatment is delivered in order to incorporate the social and cognitive challenges typical of autism (Crane et al., 2019; Mason et al., 2019). Needs and preferences among autistic adults include the utilization of written communication, elimination of metaphors from treatment materials, reduction of sensory overload as well as a stronger focus on behavioral rather than cognitive mechanisms within the intervention (Hourston & Atchley, 2017; Nicolaidis et al., 2015; White et al., 2018).

Mindfulness-based stress reduction (MBSR) is a structured group intervention that aims to provide effective and sustainable stress-coping strategies (Kabat-Zinn, 1982; Kabat-Zinn, 2013). MBSR seeks to cultivate mind-body awareness, attitudes of acceptance and regulation of responses to stressors through experiential learning (“learning by doing”), rather than through high-level cognitive strategies (Blacker et al., 2009). MBSR has previously shown to reduce perceived stress and improve symptoms of anxiety and depression in both clinical and non-clinical samples (Keng et al., 2011). A small number of studies have investigated mindfulness-based interventions for autistic adults with preliminary results indicating improved well-being and quality of life (Cachia et al., 2016; Hourston & Atchley, 2017; White et al., 2018). The emerging literature covers different types of mindfulness interventions, based on mindfulness-based cognitive therapy (MCBT) (Kiep et al., 2015; Sizoo & Kuiper, 2017; Spek et al., 2013) or MBSR (Beck et al., 2020; Braden et al., 2022). Due to adjustments in the treatment contents and/or delivery, the interventions vary in different studies. This proliferation of forms of intervention leads to different treatment protocols (Cachia et al., 2016), and variability in teacher training levels and competency, potentially complicating future dissemination and implementation into clinical settings (Howlin, 2021). However, the standard MBSR program is internationally well-known, and widely available. It comprises of a structured intervention manual, as well as stepwise teacher training and supervision, supporting implementation with fidelity. To increase external validity and to facilitate sustainable future implementation, studies following the traditional MBSR program’s contents, while adjusting the delivery to the autistic adults’ needs, are warranted (Hourston & Atchley, 2017). Thus far, some pilot studies have applied this approach with indications of improvements in positive outlook, as well as an improvement in disability- and mental health-related qualities of life, for autistic adults with very high educational level (Beck et al., 2020) and those recruited in a research center context (Braden et al., 2022). Studies in an outpatient context, recruiting from the regular patient base, and employing ordinary staff members as MBSR teachers, are warranted to inform about MBSR’s feasibility in a clinical setting.

The primary aim of this study was to thus evaluate the feasibility (completion rate) and acceptability (perception of treatment credibility and expectation of improvement) of the MBSR program for intellectually able autistic adults in an outpatient habilitation services (disability health care) context. A secondary aim was to evaluate preliminary effectiveness including perceived stress and difficulties with coping.

## Method

### Study setting and design

An open feasibility study was conducted in a clinical outpatient setting at two adult outpatient habilitation centers in Stockholm, Sweden. These habilitation centers are part of Habilitation and Health, Region Stockholm and provide outpatient health care supporting functioning in everyday life and active participation within communities of people with enduring disabilities. Data was collected using self-report questionnaires conducted pre- and post-intervention from seven MBSR groups between 2016 and 2018. Group sizes ranged from 5 to 10 participants.

Written informed consent was collected from all participants, and the study was approved by the Regional Ethics Committee in Stockholm, Sweden (2016/115-31/4).

### Participant eligibility and enrollment

The aim was to include a referred sample of autistic adults with documented autism diagnosis without intellectual disability, typically presenting in an outpatient clinical context. Therefore, commonly co-occurring neurodevelopmental (e.g. attention-deficit/hyperactivity disorder (ADHD)) or psychiatric disorders (e.g. depression, anxiety) were not an exclusion criterion. The participants were adults diagnosed with an autism spectrum disorder according to the *Diagnostic and Statistical Manual of Mental Disorders* (4th ed.; DSM-IV; American Psychiatric Association, 2000), the *Diagnostic and Statistical Manual of Mental Disorders* (5th ed.; DSM-5; American Psychiatric Association, 2013), or the *International Statistical Classification of Diseases and Related Health Problems–*Tenth Edition (ICD-10; World Health Organization [WHO], 1993) and without intellectual disability (psychometrically determined intelligence quotient (IQ) > 70). Participants were verbally fluent in Swedish and willing to participate in a group intervention adapted to autistic adults’ needs. Exclusion criteria were diagnosed intellectual disability, severe mental illnesses (e.g. psychosis or acute risk for suicidal behaviors), illegal drug use or alcohol use disorder in the last 3 months or adverse psychosocial circumstances (e.g. being homeless), precluding active intervention participation.

Participants were informed about the study both orally and in writing by clinic staff and asked about their interest to participate prior to being scheduled for an eligibility assessment. The assessment was conducted by an experienced clinical psychologist in co-operation with an MBSR teacher and addressed the ability and motivation to take part in a group setting, as well as current mental health status. Mental health was assessed using the Montgomery–Åsberg Depression Rating Scale (Montgomery & Asberg, 1979) in combination with a semi-structured interview conducted by an experienced clinical psychologist.

### The MBSR intervention

The MBSR intervention (Blacker et al., 2009; Kabat-Zinn, 1982) included eight two and half-hour weekly group sessions as well as one all-day silent retreat. The sessions 1 to 5 teach meditations and cultivate non-judgmental awareness while the sessions 6 to 8 focus on application of mindful awareness and the new coping skills in everyday situations.

#### Community involvement in the intervention

Before onset of the study, a few MBSR groups were delivered to obtain the autistic adults’ perspectives and needs for adjustments to delivery. The participants provided feedback spontaneously during the intervention, and their feedback was also gathered at the end of the intervention, using a semi-structured group discussion as method. The group discussion focused on lived experiences of MBSR, participants’ gains from the intervention, and supporting and hindering factors for the MBSR intervention and for continued mindfulness practice. The implemented adjustments (to reduce hindering factors) are described in the next section. During the study, seven groups were included, and each of these also gave their feedback using questionnaires (see below in the Section “Measures/Feasibility in an outpatient setting) and group discussions at the end of the intervention. This feedback largely confirmed the acceptability of the implemented adjustments.

#### Adjustments in delivery of the MBSR program for autistic adults

The program contents used in this study follows the original MBSR program (Blacker et al., 2009; Kabat-Zinn, 1982). However, since limited consideration of autistic adults’ needs has been reported to hinder access to treatment (Adams & Young, 2021) and, in accordance with other studies (Cachia et al., 2016; Hourston & Atchley, 2017; White et al., 2018), adjustments regarding the delivery of the MBSR intervention were discussed and agreed upon within the project group consisting of researchers and MBSR teachers. Adjustments, aiming at facilitating participation for autistic adults, were described in an amendment to the MBSR manual and included the following. (1) Group size: the number of participants was limited to a maximum of 10 patients per group. (2) Physical environment: patients who wanted to avoid waitroom environment could enter the intervention room directly upon arrival to the clinic. A designated spot for each participant was arranged beforehand. To address sensory sensitivities, potentially disturbing elements in the room were removed, the lighting was adjusted, and the participants were recommended not to wear perfumes. For the breaks, participants could choose to stay in the room or to go outside. (3) Session structure and teacher–participant communication: at session one, a detailed agenda was written on the white board to enhance predictability of the procedure. In the following sessions, the theme of the day was written on the white board to support orientation and focus. MBSR teachers used concrete communication free from metaphors. For example, instructions such as “breathe through your body parts” were specified as “concentrate on the feeling in your (e.g.) right arm; does it feel warm, cold, heavy . . .,” and so on. (4) Silent retreat day: the retreat day in silence was 5.5 h, and the participants were informed about the possibility to receive individual support during the retreat if necessary. (5) Homework: if requested by the participants, the MBSR teachers facilitated planning of the voluntary homework assignments. The MBSR teachers ensured that completion to homework did not colligate with a sense of success or failure. (6) Workbook and materials: the text sections in the workbook were shortened to make them easier to follow for autistic adults. The most central exercises were provided as audio files to facilitate the practice in-between group sessions and continued training after the group program. Length of meditations was shortened from the original program of approximately 45 to 10–25 min. The amendment to the MBSR manual (in Swedish only), describing the adjustments, can be obtained from the corresponding author on presentation of level one MBSR teacher certificate.

### Treatment fidelity

Each group was guided by two MBSR teachers, who were also regular staff members at the habilitation clinics, with experience in working with autistic adults. For each of the seven included groups, at least one teacher had maintained several years of formal meditation practice. At least one of the teachers was trained to level one of teacher training, following international standards (iminetwork.org), with the other at least under training toward level one. Both teachers were trained by Center for Mindfulness Sweden following the MBSR teacher training curriculum as defined by the Center for Mindfulness in medicine, health care, and society at the University of Massachusetts Medical School. The MBSR teachers also received regular supervision from a highly experienced (level 3) certified international teacher trainer in MBSR currently active at the Center for Mindfulness Sweden.

### Measures

#### Demographic variables and background information

Demographic data were collected through the questionnaire “Current Life Situation” (Hirvikoski et al., 2009). Educational level was first categorized into three categories of highest level achieved (compulsory school (9 years or less); upper secondary school; academic degree). However, since there were few participants with the educational level of compulsory school or less, a dichotomous categorization (academic/university degree yes/no) was applied. Occupation was categorized to (1) working or studying; (2) supported employment or day activity center; or (3) not working or studying, including long-term sick leave, unemployment, and disability pension. Specific demographic data on participants’ race and ethnicity were not recorded, since registration of race or ethnicity is not a recommended procedure in Sweden. The MBSR teachers had access to the participants’ clinical records in order to verify the autism diagnosis and reported co-occurring psychiatric diagnoses, which is routinely performed at habilitation centers.

### Feasibility in an outpatient setting

#### Treatment completion

According to a benchmark defined a priori, participants needed to attend six or more out of the total nine classes (eight sessions and the all-day retreat) to be considered completers. This benchmark was set based on our clinical experience and previous studies on attrition in psychiatric outpatient services (Mazzotti & Barbaranelli, 2012).

#### Adverse events/serious adverse events

Adverse events (AE) were defined as any spontaneously reported inconveniences, and serious adverse events (SAE) were defined as events that required hospital care. AE and SAE during the course of the study were reported to the study clinicians and consecutively recorded in the participant’s individual case report form. The potential association of the AE and SAE to the intervention was evaluated by the project group consisting of researchers with expertise in this population together with the MBSR teachers, after the data collection was finished.

#### Treatment credibility

Treatment credibility and expectations of improvement following the intervention were assessed using an adjusted version of the Treatment Credibility Scale (TCS; Borkovec & Nau, 1972). It contains five items that are scored on a 10-point scale (1–10) with increasing values indicating higher treatment credibility: *How much sense does MBSR make based on your previous experiences; how sure are you that this kind of course will be successful in increasing your mindful awareness; would you recommend this course to a friend with the same diagnosis as yourself; do you think this type of course is a good way to learn about mindful awareness; after finishing the course, how much do you expect to have learnt about mindful awareness and stress reduction?* Since initial perception of treatment credibility may be associated with post-treatment outcomes (Constantino et al., 2018), we administered the TCS at baseline. However, to measure the participants’ perception of the intervention’s credibility after their firsthand experience of the entire MBSR program, we also administered the TCS post-intervention. The TCS has been recently used in another trial for autistic adults (Hidalgo et al., 2022). In the current data set, the internal consistency of the scale was Cronbach’s α = 0.87.

### Secondary outcomes

Stress was measured using the 14-item Perceived Stress Scale (PSS), scored on a 5-point Likert-type scale (0–4) where higher points indicate the higher perceived stress (Cohen et al., 1983). The 14-item version was chosen to use the previously identified two-factor solution, measuring perceived difficulties with coping (e.g. *Confidence in own ability to handle personal problems; Dealing successfully with everyday problems*) and perceived distress (e.g. *Feeling nervous and stressed; Feeling out of control*) (Hewitt et al., 1992). In this data set, the internal consistency of the PSS was Cronbach’s α = 0.85.

Symptoms of anxiety and depression were assessed using the Hospital Anxiety and Depression Scale. Fourteen statements scored on a 4-point Likert-type scale (0–3), where higher points indicate more anxiety and depressive symptoms. In addition, the 7-item sub-scales for anxiety and depression were analyzed separately (Lisspers et al., 1997; Zigmond & Snaith, 1983). A subscale score ⩾8 represents clinical levels of anxiety or depression symptoms (Brennan et al., 2010). In this data set, the internal consistency of the whole scale was Cronbach’s α = 0.89.

Overall life satisfaction was measured using the Satisfaction With Life Scale, five statements scored on a 7-point Likert-type scale (1–7) where higher points indicate more satisfaction with life (Diener et al., 1985). In this data set, the internal consistency of the scale was Cronbach’s α = 0.80.

Mindfulness was assessed using the Mindful Attention Awareness Scale, consisting of 15 statements and scored on a 6-point Likert-type scale (1–6), where higher scores indicate higher levels of mindful awareness (Brown & Ryan, 2003). In this data set, the internal consistency of the scale was Cronbach’s α = 0.83.

For acceptance of the autism diagnosis, a modified version of the Acceptance and Action Questionnaire (Backman et al., 2018; Bond et al., 2011) was administered, consisting of seven statements (e.g. “My diagnosis makes it hard for me to pursue a life that I could value” and “I feel uncomfortable about my diagnosis”) scored on a 7-point Likert-type scale (1–7), where higher points indicate less acceptance of the autism diagnosis. In this data set, the internal consistency of the scale was Cronbach’s α = 0.82.

### Statistical analysis

The total number of outliers across all measures was *n* = 7, and none of these outliers were extreme. Therefore, no action was performed to manage them, and hence they were not excluded from statistical analysis. Demographic data and background variables for completers were compared to non-completers, using Student’s *t*-test for continuous variables and Chi-square test or Fisher’s exact test for categorical variables. Preliminary effectiveness was analyzed using paired samples *t*-tests from pre- to post-intervention for all completers with complete data (*per protocol*). Effect size was assessed using Cohen’s *d* (Cohen, 1988).

## Results

**Figure 1.**
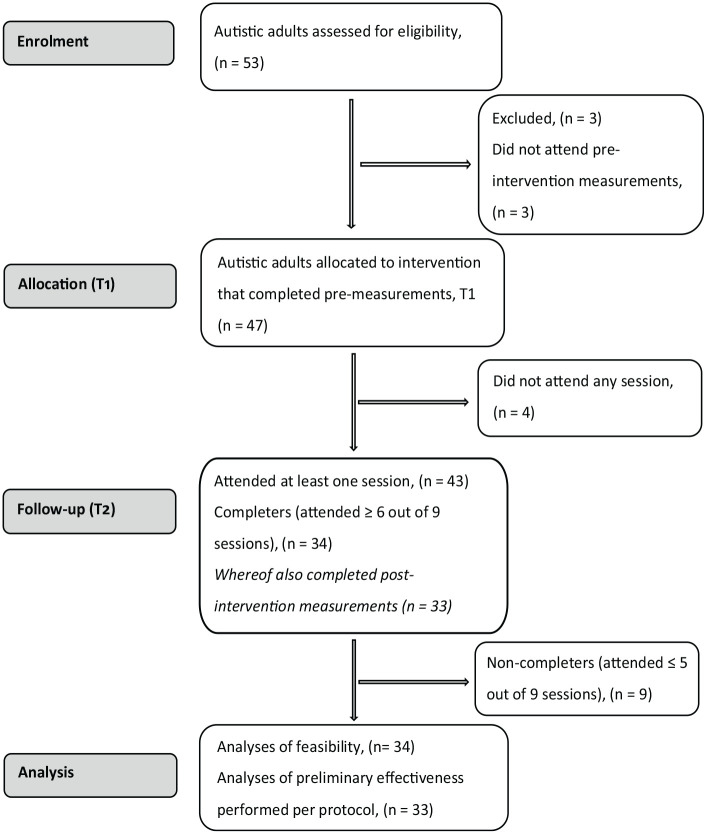
Flowchart of the study participants from recruitment to analyses.

The Consolidating Standard of Reporting Trials (CONSORT) checklist for feasibility studies is provided as Supplementary Materials, and a CONSORT flowchart of participants is presented in Figure 1.

The participants’ mean age was 36.2 years (standard deviation (SD) = 12.8). *N* = 16 (37.2%) of the participants were diagnosed with at least one additional neurodevelopmental disorder (mainly ADHD) and *n* = 26 (60.5%) with at least one additional psychiatric disorder. Most of the participants (*n* = 29, 67.4%) were not in a partner relationship and 20 (46.5%) reported being outside of any (regular or supported) work or education. Demographic- and background data are presented in detail in Table 1.

**Table 1.** Sample characteristics for participants who started the intervention.

Participants with autism	*n* = 43
Age (years)	M = 36.2, SD = 12.8 Range = 20–65
Gender	
Female	*n* = 21 (48.8%)
Male	*n* = 21 (48.8%)
Missing	*n* = 1 (2.3%)
Co-occurring diagnoses	
At least one additional neurodevelopmental disorder (mainly ADHD)	*n* = 16 (37.2%)
At least one additional psychiatric disorder (mainly depression or anxiety disorders)	*n* = 26 (60.5%)
Relationship and civil status	
Married/partner	*n* = 12 (27.9%)
Single/not married/divorced	*n* = 29 (67.4%)
Missing	*n* = 2 (4.7%)
Education, highest level accomplished	
No academic/university degree^a^	*n* = 29 (67.4%)
Academic/university degree	*n* = 12 (27.9%)
Missing	*n* = 2 (4.7%)
Occupation	
Working or studying^b^	*n* = 14 (32.6%)
Supported employment^c^	*n* = 7 (16.3%)
Not currently working or studying^d^	*n* = 20 (46.5%)
Missing	*n* = 2 (4.7%)

ADHD: attention-deficit/hyperactivity disorder.

a Includes compulsory school (9 years or less) and upper secondary school.

b Includes employment, self-employment and studies, parental leave, and part- or full-time.

c Includes Day Activity Center, trainee job/internship, and so on.

d Includes unemployment, long-term sick leave, disability pension, and so on.

### Feasibility

#### Treatment completion

Thirty-four participants out of 43 attending at least one session (79%) took part in at least 6 classes out of 9 and were considered intervention completers (Figure 1). None of the background and demographic variables at baseline (depicted in Table 1) differed between the completers (*n* = 34) and the non-completers (*n* = 9) (all *p*-values > 0.10, data not shown). Likewise, no differences between the completers and the non-completers were observed in perceived treatment credibility or expectation (TCS) or any of the secondary outcome measures (PSS, Hospital Anxiety and Depression Scale (HADS), Mindful Attention Awareness Scale (MAAS), Acceptance and Action Questionnaire (AAQ) autism, or Satisfaction With Life Scale (SWLS)) at baseline.

#### Treatment credibility

**Figure 2.**
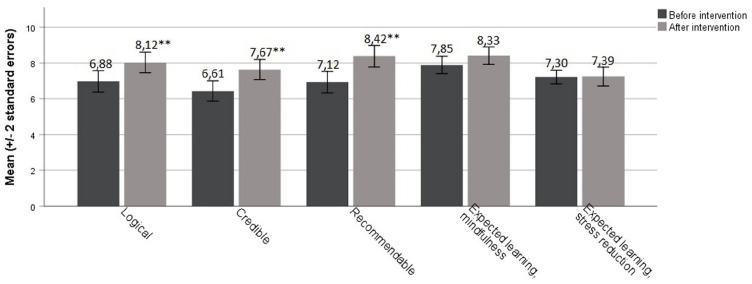
Pre- and post-assessment scores of treatment credibility and expectations using the Treatment Credibility Scale. *N* = 34 with competed data before and after intervention; ***p ⩽ 0.001.

The participants reported increased treatment credibility from pre- to post-intervention (the mean total score difference of the Treatment Credibility Scale (*t*(32) = −3.71, *p* = 0.001, *d* = 0.61; at post-intervention total TCS score M = 7.99, SD = 1.45, range = 4.6–10.0)). Specifically, the participants reported better treatment credibility regarding three of the five items: “How much sense does MBSR make based on your previous experiences?” (*t*(32) = −3.60, *p* = 0.001, *d* = 0.67); “How sure are you that this kind of course will be successful in increasing your mindful awareness?” (*t*(32) = −3.48, *p* = 0.001, *d* = 0.62); “Would you recommend this course to a friend with the same diagnosis as yourself?” (*t*(32) = −4.02, *p* < 0.001, *d* = 0.74). Mean and standard error for all five items, as well as pre- and post-intervention scores, are presented in Figure 2.

#### AEs and SAEs

Three AEs were reported by three participants during or after the intervention. Two of the events were related to stressful life events (e.g. regarding housing situation) while one could be related to the intervention (increased anxiety during one session; the participant was able to see the session through after receiving individual support). No SAEs were reported.

### Preliminary effectiveness

Preliminary effectiveness was calculated based on the responses of *n* = 33 participants, since one of the 34 completers did not fill in post-intervention measures. Perceived stress was reduced from pre- to post-intervention in the PSS distress subscale (*t*(32) = 3.35, *p* = 0.002, *d* = 0.48). The participants also reported better ability to cope with stressors after the intervention (*t*(32) = 2.52, *p* = 0.017, *d* = 0.43).

In addition, participants reported reduced symptoms of anxiety (*t*(32) = 2.30 *p* = 0.028, *d* = 0.33) and depression (*t*(32) = 2.43, *p* = 0.021, *d* = 0.30). At baseline, 75.8% of the completers scored above the clinical cut-off (⩾8) for HADS anxiety, while the corresponding figure at post-intervention was 57.6%. For HADS depression, 51.5% of the participants scored above the clinical cut-off at baseline, and 48.5% at post-intervention. No significant changes were observed for mindful attention awareness, satisfaction with life, or acceptance of the autism diagnosis. The results of preliminary effectiveness are presented in Table 2.

**Table 2.** Pre- and post-assessment scores of secondary outcome measures.

Measure	Pre M (SD)	Post M (SD)	*t*(32)	*p*	Cohen’s *d*
Perceived Stress Scale					
PSS, subscale coping	7.61 (2.85)	6.42 (2.73)	2.516	***p*** **=** **0.017**	0.43
PSS, subscale distress	18.09 (4.69)	15.79 (4.82)	3.350	***p*** **=** **0.002**	0.48
MAAS	3.65 (0.74)	3.63 (0.79)	0.180	*p* = 0.858	0.03
Hospital Anxiety and Depression Scale					
HADS, subscale anxiety	10.58 (4.24)	9.18 (4.34)	2.300	***p*** **=** **0.028**	0.33
HADS, subscale depression	8.36 (4.44)	7.03 (4.40)	2.432	***p*** **=** **0.021**	0.30
AAQ—autism	24.85 (7.80)	23.64 (8.50)	0.971	*p* = 0.339	0.18
SWLS	14.79 (5.95)	15.79 (6.03)	−1.450	*p* = 0.157	0.17

M: mean value; SD: standard deviation; PSS: Perceived Stress Scale; MAAS: Mindful Attention Awareness Scale; HADS: Hospital Anxiety and Depression Scale; AAQ: Acceptance and Action Questionnaire modified to assess acceptance of autism diagnosis; SWLS: Satisfaction With Life Scale.

N = 33 with data at both pre- and post-intervention; p-values in bold indicate the statistical significance (p < 0.05).

## Discussion

This study, evaluating the feasibility and preliminary effectiveness of MBSR for autistic adults in an outpatient context, found that the feasibility benchmark for treatment completion was met. Treatment credibility improved from pre- to post-intervention and no SAEs related to the intervention were reported. Moreover, participants’ reports on preliminary effectiveness showed a reduction in perceived stress, and increased skills in coping with stress from pre- to post-intervention. Also, symptoms of anxiety and depression were reduced, while the quality of life, participants’ mindfulness skills, and acceptance of autism diagnosis were unchanged. Treatment completion rate was 79%.

To the best of our knowledge, this is the first study to evaluate treatment completion in MBSR for autistic adults in an outpatient clinical context, and therefore, comparisons to previous studies are difficult. Generally, patients receiving different kinds of psychiatric services have shown completion rates ranging from 18% to 74% (Mazzotti & Barbaranelli, 2012), and so, in comparison, the completion rate in this study may be considered good. Interestingly, this acceptable completion rate was reached despite the difficulties regarding occupation and psychiatric comorbidity among the participants. For example, almost half of the participants were outside of any (regular or supported) employment, although their educational level was close to the general population in Sweden, where approximately 30% have a university degree (Statistics Sweden, 2022). Moreover, a majority of the participants had co-occurring neurodevelopmental or other psychiatric diagnoses. Some previous studies have excluded participants with co-occurring neurodevelopmental disorder (Kiep et al., 2015; Spek et al., 2013), while others have, like us, aimed at including representative samples for autistic adults without intellectual disability and therefore have not excluded patients with psychiatric co-occurrence (Braden et al., 2022). This study sample and good completion rate reached for these participants may hence also generalize rather well to autistic adults presenting at similar outpatient health care services.

The participants found the intervention credible and logical and would recommend it to other autistic adults. This is in line with a previous pilot study demonstrating high acceptability of standard MBSR among autistic adults with high educational level (>80% of participant having academic exam) (Beck et al., 2020). The level of treatment credibility was on a par with another recent study using the same assessment instrument (TCS), although evaluating a first-line psychoeducational intervention for autistic adults (Hidalgo et al., 2022). These results are encouraging given the frequent reports of difficulties in health care encounters among autistic adults (Camm-Crosbie et al., 2019; Coleman-Fountain et al., 2020; Mason et al., 2019). The experiential learning incorporated in MBSR, focusing on adaptive coping skills rather than the more cognitive focus of classical CBT (White et al., 2018), together with described adjustments in delivery may have contributed to the perceived treatment credibility. Future qualitative studies on participants’ experiences of MBSR practicing would provide a more detailed understanding of treatment credibility and acceptability for autistic adults.

The preliminary effectiveness measures showed medium-sized effects in the reduction of perceived stress and perceived difficulties with coping, from pre- to post-intervention. These results are promising, given the commonality of high stress in this patient population (Hirvikoski & Blomqvist, 2015; Moseley et al., 2021). Improvements in coping skills could increase resilience in autistic adults and potentially reduce the risk of developing mental health conditions and the need of specialized mental health services. However, the results should be interpreted cautiously given the open study design, and as this is the first study to explicitly evaluate the association between MBSR and subjective stress and coping for autistic adults. However, in line with studies on other mindfulness interventions for autistic adults (Kiep et al., 2015; Sizoo & Kuiper, 2017; Spek et al., 2013), we also observed a small reduction in symptoms of anxiety and depression from pre- to post-intervention. The depression symptoms were reduced from clinical range at baseline to subclinical range post-intervention, according to previously defined cut-off limits (Brennan et al., 2010). These promising results warrant further evaluation in larger randomized controlled trials, since depression and anxiety are the most common psychiatric co-occurrences (Hollocks et al., 2019; Linden et al., 2022), and psychiatric comorbidity is an important risk factor for suicidal behaviors in autistic adults (Hirvikoski et al., 2020), while the evidence base for mental health interventions for autistic adults is still poor (Linden et al., 2022). In line with another MBSR study including autistic adults (Beck et al., 2020), mindful awareness did not significantly change from pre- to post-intervention, highlighting the need of further studies on potential intervention mechanisms. Improved self-reflection and emotion regulation have been suggested as candidate mechanisms for mindfulness interventions for autistic adults (Pagni et al., 2020) and could thus be assessed in future studies regarding intervention mechanisms.

The results of this study need to be interpreted from the perspective that the MBSR teachers followed the original program’s contents and received continuous supervision to ensure high treatment fidelity. However, adaptations in the delivery of the intervention were made to support active participation for the autistic adults (Crane et al., 2019; Lipinski et al., 2021; Mason et al., 2019). This approach improves replicability and generalizability, and hence facilitates later dissemination and implementation of the intervention into clinical practices, possibly alleviating current lack of acceptable and accessible interventions. Adult services struggle with providing adequate care for autistic adults (Anderson et al., 2022; Camm-Crosbie et al., 2019; Crane et al., 2019; Nicolaidis et al., 2015), while group interventions have the advantage of supporting multiple participants simultaneously, hence possibly improving health care accessibility (Adams & Young, 2021).

### Limitations

This study was an open feasibility study, limiting any definite conclusions about effectiveness. Second, the self-rating questionnaires used in this study were not adapted for autistic individuals which could have reduced the validity or responsiveness to change of these measures (Wigham & McConachie, 2014). Third, the results comparing demographic and background variables for completers versus non-completers should be interpreted with caution due to small sample sizes, especially in the non-completer group. Moreover, the definition of AEs as “any spontaneously reported inconvenience” may be associated with failing to capture some transient distress and negative impacts, which are known to be associated with mindfulness-based programs as often as with other psychological interventions (Britton et al., 2021). Finally, although MBSR is based on experiential learning, the treatment adherence to the homework assignments was not systematically evaluated in this study.

## Conclusion

The current feasibility study indicated that MBSR is a feasible and acceptable intervention for autistic adults presenting in an outpatient context. The results can generalize to similar clinical contexts, meeting needs of resilience-building preventive interventions for autistic adults. The effectiveness of MBSR for autistic adults must be further corroborated in randomized clinical trials.

## Supplemental Material

sj-docx-1-aut-10.1177_13623613231172809 – Supplemental material for Mindfulness-based stress reduction for autistic adults: A feasibility study in an outpatient context Click here for additional data file. Supplemental material, sj-docx-1-aut-10.1177_13623613231172809 for Mindfulness-based stress reduction for autistic adults: A feasibility study in an outpatient context by Hanna Agius, Anne-Kristina Luoto, Anna Backman, Carina Eriksdotter, Nitya Jayaram-Lindström, Sven Bölte and Tatja Hirvikoski in Autism
